# Supplementary figures and images for: Multimodal Comparison of Diabetic Neuropathy in Aged Streptozotocin-Treated Sprague–Dawley and Zucker Diabetic Fatty Rats
Source: Biomedicines. 2022 Dec 22;11(1):20. doi: 10.3390/biomedicines11010020 (PMC9855818; doi:10.3390/biomedicines11010020)

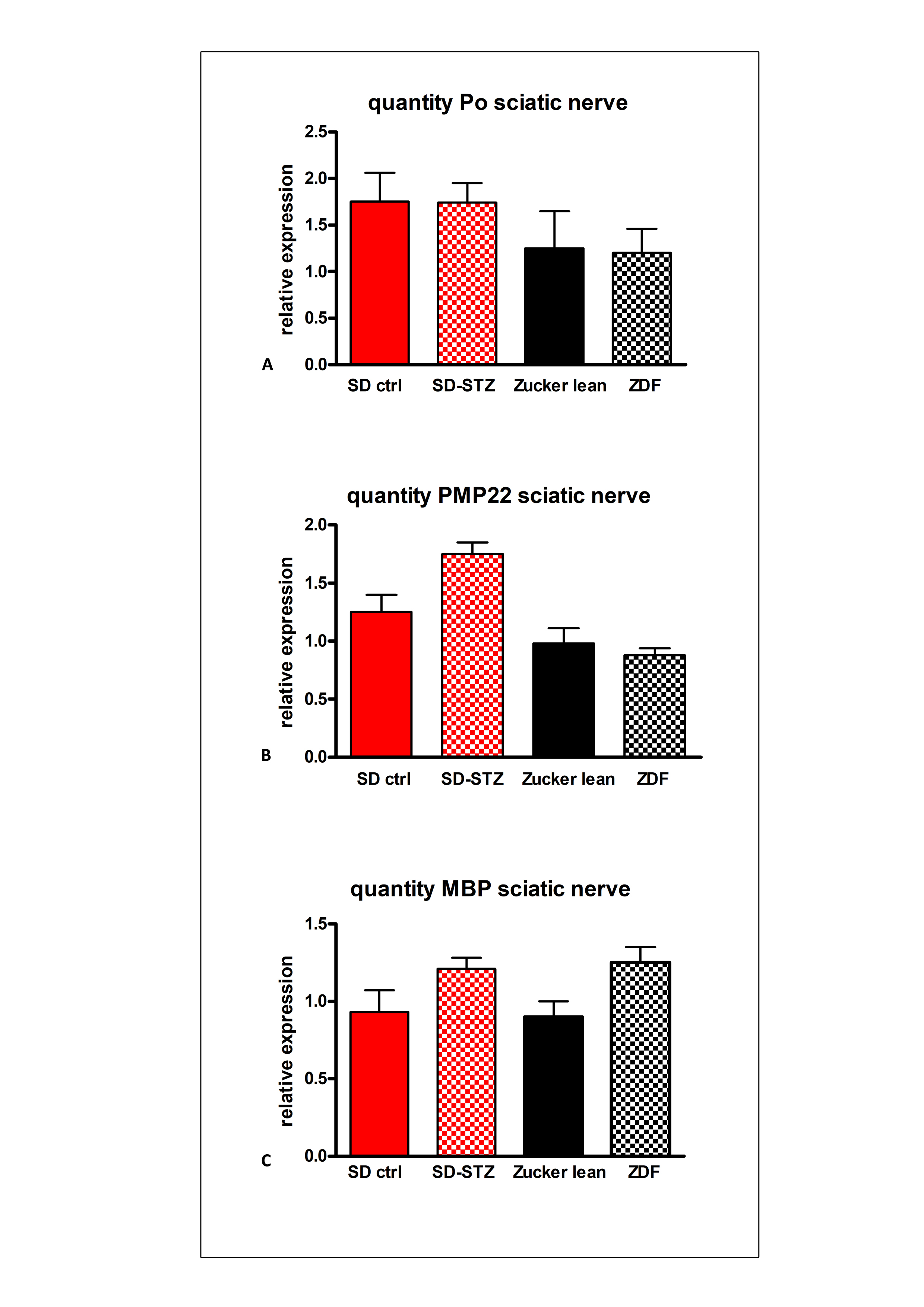

Supplement: Supplementary file 1 [file biomedicines-11-00020-s001.zip › biomedicines-2014860-supplementary.jpg]
